# Syndrome of uremic encephalopathy and bilateral basal ganglia lesions in non-diabetic hemodialysis patient: a case report

**DOI:** 10.1186/s12882-018-1174-0

**Published:** 2018-12-19

**Authors:** Wen-Yu Gong, Shan-Shan Li, Zong-Chao Yu, Hong-Wei Wu, Liang-Hong Yin, Li-Fan Mei, Fan-Na Liu

**Affiliations:** 1 Division of Nephrology, Department of medicine, the First Affiliated Hospital, Jinan University, Guangzhou, 510630 Guangdong China; 2 Division of Nephrology, Department of medicine, Zhongshan Boai Hospital, Zhongshan, 528400 Guangdong China

**Keywords:** Uremic encephalopathy, Bilateral basal ganglia lesions, Non-diabetic, Hemodialysis

## Abstract

**Background:**

Uremic encephalopathy (UE), a toxic metabolic encephalopathy, is an uncommon complication resulting from endogenous uremic toxins in patients with severe renal failure. UE syndrome can range from mild inattention to coma. The imaging findings of UE include cortical or subcortical involvement, basal ganglia involvement and white matter involvement. The basal ganglia type is uncommon, although previous cases have reported that Asian patients with diabetes mellitus (DM) are usually affected.

**Case presentation:**

A 32 year-old woman with a history of non-diabetic hemodialysis for 3 years suffered from severe involuntary movement, and brain magnetic resonance imaging showed symmetrical T2-weighted imaging (T2WI) and T2/fluid-attenuated inversion recovery (T2FLAIR) hyperintense nonhemorrhagic lesions in the bilateral basal ganglia. She was diagnosed with UE as syndrome of bilateral basal ganglia lesions, due to a combined effect of uremic toxins and hyperthyroidism. After treatment with high frequency and high flux dialysis, hyperbaric oxygen therapy and declining parathyroid hormone, the patient achieved complete remission with normal body movement and was discharged.

**Conclusion:**

UE with basal ganglia involvement is uncommon, although generally seen in Asian patients with DM. Our case reported a hemodialysis patient that had non-diabetic UE with typical bilateral basal ganglia lesions, presenting with involuntary movement.

## Background

Uremic encephalopathy (UE) is an uncommon metabolic disorder syndrome, which is characterized by reversible neurological symptoms of acute or subacute episodes. It generally occurs in patients with acute kidney injury or severe chronic kidney disease, which may result from multiple metabolic derangements [1, 2]. According to previous studies, UE, a type of toxic metabolic encephalopathy, is a complication of endogenous uremia, and is usually seen in severe renal failure patients. UE presents with symptoms ranging from mild inattention to coma, and can also accompanied by sleep disorders, headache, dysarthria, gait disorders, and less frequently by extrapyramidal movements such as involuntary movement, chorea and bradykinesia [2–4].

According to previous studies, imaging findings of UE can be classified into three types: 1) cortical or subcortical involvement, 2) basal ganglia involvement, and 3) white matter involvement [2]. UE with cortical or subcortical involvement is more common and may develop in any uremic patient, with no effect of diabetes mellitus (DM) [2, 5]. The basal ganglia type is uncommon, and previous studies have reported that Asian patients with DM are usually affected [2, 6, 7]. Finally, the white matter type is rare and is limited to case reports [5, 8]. Acute bilateral basal ganglia lesions in the background of dialysis constitute a unique syndrome, particularly in diabetic uremic patients [9].

Here, we report a case of a non-diabetic dialysis patient with UE and typical acute bilateral basal ganglia lesions, along with a literature review.

## Case presentation

**Fig. 1:**
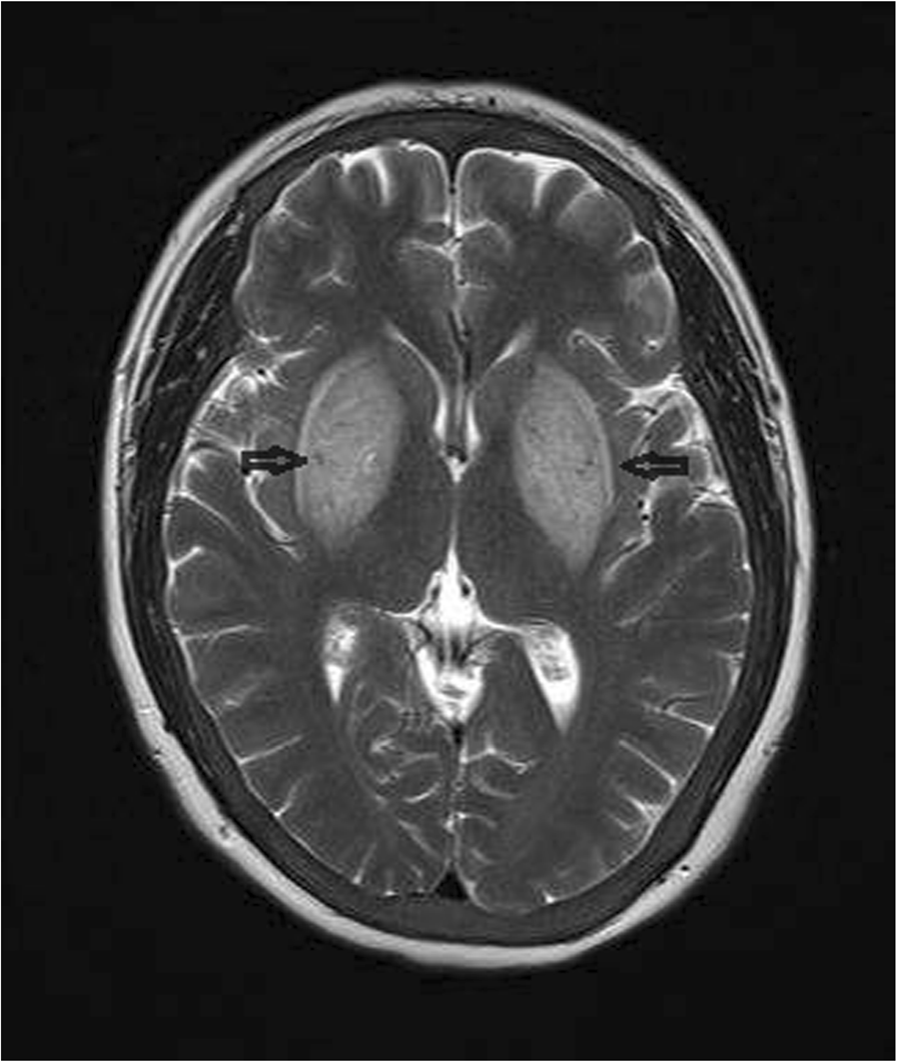
Brain MRI performed upon admission. T2WI showed symmetrical hyperintense non-hemorrhagic lesions in the entire bilateral basal ganglia (arrows), and corona radiata lesions showing mild diffusion restriction

**Fig. 2:**
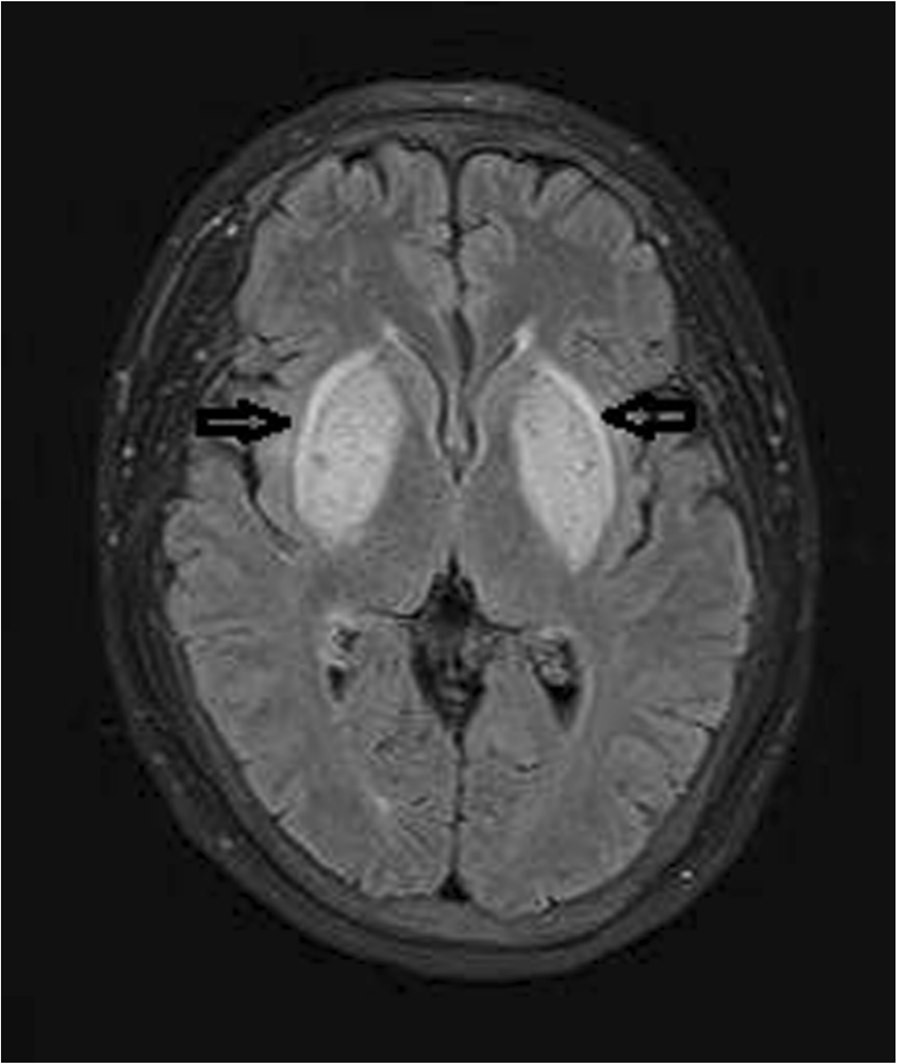
Brain MRI performed upon admission. T2FLAIR showed symmetrical hyperintense non-hemorrhagic lesions in the entire bilateral basal ganglia (arrows) and corona radiata lesions showing mild diffusion restriction

A 32-year-old female with non-diabetic chronic kidney disease was on regular hemodialysis for 3 years, via a right forearm arteriovenous fistula. The patient was admitted to our department due to involuntary movement for 5 days. Starting 5 days prior to admission, the patient’s shoulder and neck displayed a resting tremor, which became increasingly severe, with the limbs beginning to move uncontrollably. The patient’s vital signs were stable, with no complaints of headache, fever, blurred vision or mental disorder. Myodynamic examination and deep tendon reflexes in both legs were normal, and the Babinski reflexes were suspiciously positive. Significant fluctuation of blood creatinine levels (predominantly due to inadequate dialysis), along with altered hyperthyroidism [intact parathyroid hormone (iPTH) levels of almost 3200 pg/mL], were reported 1 week ago with no accompanying history of hypertension, DM, respiratory tract infection, fever, stoke, liver disease, hypoxia or toxic fume exposure. Brain magnetic resonance imaging (MRI) was performed 5 days after the onset of symptoms in the local hospital, and showed symmetrical T2-weighted imaging (T2WI; Fig. 1) and T2/fluid-attenuated inversion recovery (T2FLAIR; Fig. 2) hyperintense non-hemorrhagic lesions in bilateral basal ganglia, as well as corona radiata lesions showing mild diffusion restriction. Both T1-weighted imaging (T1WI) and diffusion-weighted images (DWI) were normal.

Blood analysis immediately after admission revealed high levels of uremic toxins (urea nitrogen 25.80 mmol/L, serum creatinine 1206 μmol/L, uric acid 548 μmol/L, phosphorus 1.88 mmol/L, calcium 2.33 mmol/L,anion gap 23.9 mmol/L), and severe hyperthyroidism (iPTH 2487 pg/mL). Bicarbonate, arterial blood gas indices, hemoglobin, albumin, lactic acid, B-vitamins and liver function were all normal.

**Fig. 3:**
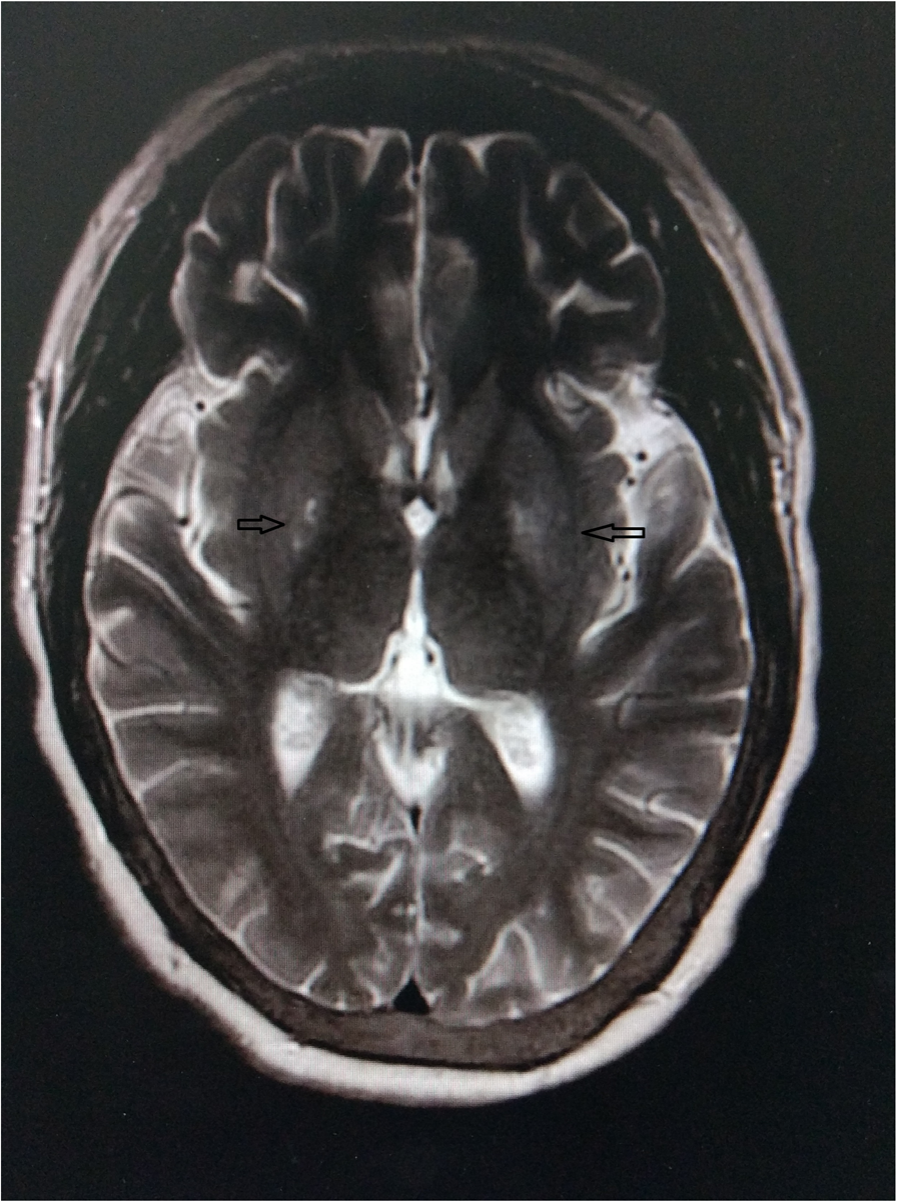
Follow-up MRI scan obtained after 14 days. T2WI revealed high signal in the bilateral basal ganglia (arrows), which were significantly weaker compared to the initial MRI signal

**Fig. 4:**
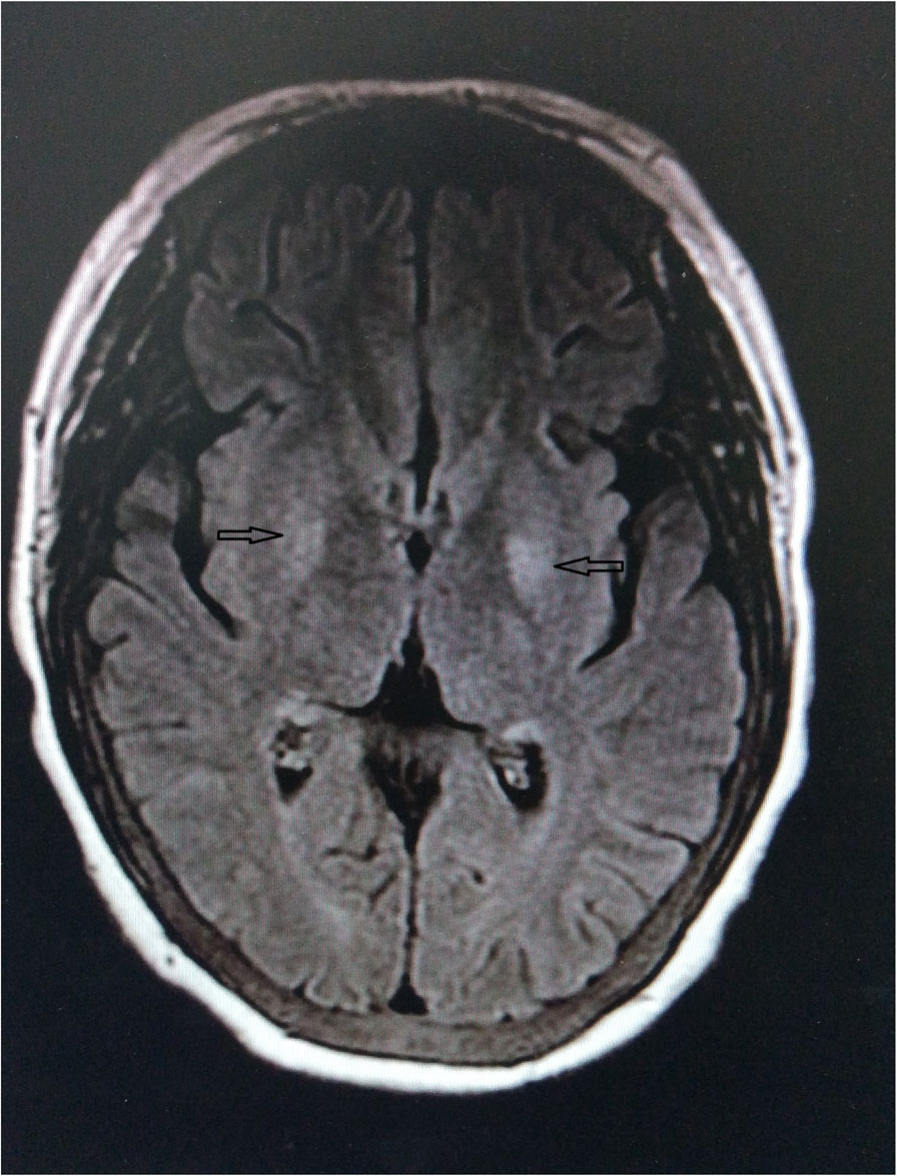
Follow-up MRI scan obtained after 14 days. T2FLAIR revealed high signal in the bilateral basal ganglia (arrows), which were significantly weaker compared to the initial MRI signal

The patient was diagnosed with UE as a symptom of bilateral basal ganglia lesions. She did not suffer from DM or other diseases such as cerebrovascular events, intoxication or hyperlactacidemia. Thus, UE was possibly due to a combined effect of uremic toxins and hyperthyroidism. The patient was treated with high frequency and high flux dialysis (4 h hemodiafiltration with APS 15 uc dialyzer, 4 times per week), hyperbaric oxygen therapy (1.5 h per day), declining parathyroid hormone (1 μg of calcitriol injection every 2 days), and treatment for symptom relief (2 mg of haloperidol, 2 mg of clonazepam and 2 mg of benzhexol administered orally twice per day). Gradually, the symptoms reduced in frequency, and the amplitude of involuntary movement decreased. Fourteen days after admission, a subsequent brain MRI revealed high signal in the bilateral basal ganglia on T2WI (Fig. 3) and T2FLAIR (Fig. 4), which were significantly weaker compared to the initial MRI signal.

**Fig. 5:**
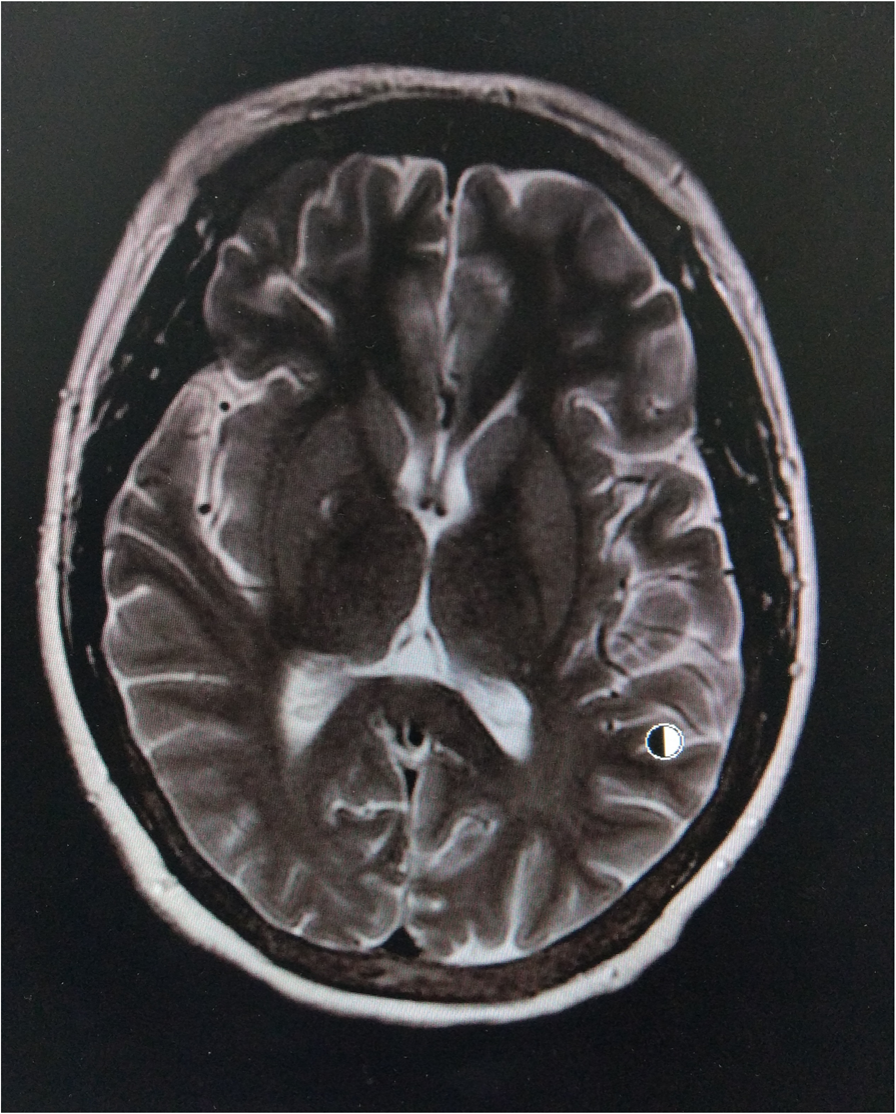
Follow-up MRI scan obtained after 1 month. T2WI showed almost complete resolution of the lesions with a slightly hyperintense signal

**Fig. 6:**
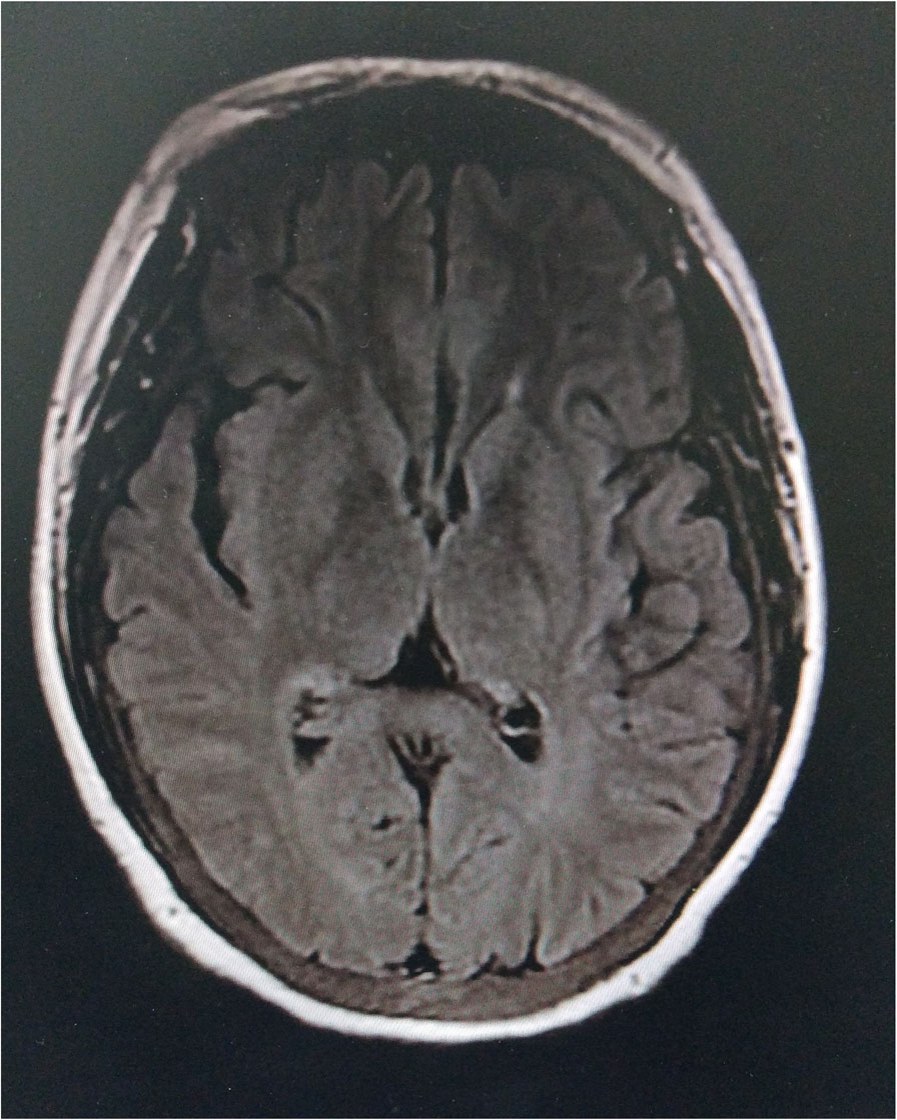
Follow-up MRI scan obtained after 1 month. T2FLAIR showed almost complete resolution of the lesions with a slightly hyperintense signal

About 1 month later, the patient achieved complete remission and restoration of normal body movement, and was discharged. Upon discharge, blood test results showed relatively stable uremic toxins (urea nitrogen 10.48 mmol/L, serum creatinine 641.5 μmol/L, uric acid 435 μmol/L, phosphorus 1.43 mmol/L, calcium 2.30 mmol/L,anion gap 15.9 mmol/L), and relieved hyperthyroidism (iPTH 1609 pg/mL). Brain MRI was performed again upon follow-up, with T2WI (Fig. 5) and T2FLAIR (Fig. 6) showing an almost complete resolution of the lesions with slightly hyperintense signal in the bilateral basal ganglia.

## Discussion and conclusions

UE is an uncommon neurological complication. As a metabolic encephalopathy, it is a clinical syndrome caused by endogenous uremia toxin. It is characterized by various acute or subacute neurological symptoms resulting from brain edema, and is usually reversible [1, 6, 10]. Clinically, UE may manifest as a series of neurological abnormalities, ranging from mild attention loss to coma, and can be further characterized by headache, sleep, articulation, and gait disorders. It is less associated with extrapyramidal movement including involuntary movement, bradykinesia, tremor, epileptic activity, chorea and Parkinson’s disease [2–4]. The clinical manifestation of our case was involuntary movement, presenting at first with resting shoulder and neck tremor which became increasingly serious, progressing to involuntary limb movement. The patient had no complaints of headache, fever, blurred vision or mental disorder.

As outlined, UE can be classified into three types, based on brain imaging results. Firstly, UE may involve cortical or subcortical regions, which is more common and may develop in any uremic patient, with no effect of DM. When affecting the cortical region, UE can cause reversible posterior leukoencephalopathy syndrome [2, 5, 6]. Secondly, UE may involve the basal ganglia, which is uncommon, although previous cases have reported that Asian patients with DM usually present with basal ganglia injury [2, 6, 7]. Finally, UE may involve white matter, which is rare subtype that is limited to case reports [5, 8]. The main radiological manifestation of UE with cortical involvement is vasogenic edema, whereas that of UE with basal ganglia involvement is both cytotoxic and vasogenic edema [11]. Some studies have reported that non-diabetic UE may lead to the development of some unusual neurologic symptoms, such as facial palsy. The imaging findings of non-diabetic UE are unique and atypical, such as only supratentorial white matter lesions, no cortex or basal ganglia involved [8]. The neuroimaging results in our case showed typical bilateral basal ganglia lesions, which were reversible and characterized by symmetrical T2WI and T2FLAIR hyperintense nonhemorrhagic lesions. Additionally, corona radiata lesions were also found, and showed mild diffusion restriction and both T1WI and DWI displaying normal signals. Usually, imaging findings such as these are reported in uremic patients along with DM, who develop acute dyskinesia in the context of a significant fluctuation in blood glucose levels (predominantly hyperglycemia) and severe uremia. However, the patient in this case did not suffer from DM and had steady blood glucose levels.

The pathogenesis of UE is complex and remains unclear, and in which neurotoxic compounds are likely to play an important role. Many factors including uremia, parathyroid hormone, metabolic acidosis, abnormal blood glucose, methylguanidine, aluminum poisoning, abnormal osmotic pressure and insufficient blood supply in the brain are considered as possible causative factors [7, 10, 12, 13]. Vanholder et al. reported that uremic toxins play a pathogenic role might through stimulating N-methyl-D-aspartate receptors and inhibiting γ-aminobutyric acid receptors. The symptoms might therefore be due to an alteration in the balance between excitatory and inhibitory amino acids and the disruption of polysynaptic pathways [14]. Furthermore, previous studies have also suggested some possible mechanisms of UE. First, a significant fluctuation in blood glucose levels (predominantly hyperglycemia) in diabetic uremic patients can increases the permeability of the blood-brain barrier. Due to cerebrovascular endothelial dysfunction (so-called dysfunction in the automatic regulation of blood vessels), uremic toxins are able to inhibit mitochondrial function, leading to destruction of the pallidum and putamen. This is seen in DM, where the basal ganglia becomes more susceptible to neurotoxic compounds [15]. Second, excessive uremic metabolic wastes may damage brain tissue, among which the basal ganglia is the most vulnerable, particularly in areas that have suffered from the obstacle of vascular autoregulation, impaired energy utilization and microangiopathy. Third, a significant decrease in cerebral oxygen consumption in uremia patients may lead to cellular edema due to disordered focal cellular metabolism [1, 5–7, 10, 11]. One study analyzed biochemical indicators of UE during the “pre-symptomatic” period. The results showed that these diabetic and nephrotic patients had metabolic acidosis and obvious biochemical indicators 2 weeks before admission. Therefore, the investigators assumed that the pre-clinical internal environment may play an important role in the pathophysiology of UE [3]. As most previous studies have reported, after renal failure has been treated and a decrease in the level of uremic toxins is achieved, clinical symptoms generally go into remission or complete recovery, and lesions seen in neuroimaging tests are lightened or disappear altogether. However, the long-term prognosis of UE remains poor according to some reports, with some patients requiring long-term intensive care on account of UE related complications, but UE itself does not. The prognosis of those basal ganglia damage or diabetic UE patients is even poorer [10, 16].

We assumed that our case may have been caused by the breakdown of the blood-brain barrier, related to the observed increase in uremic toxins, which measured as follows: urea nitrogen 25.80 mmol/L, creatinine 1206 μmol/L, uric acid 548 μmol/L, phosphorus 1.88 mmol/L, calcium 2.33 mmol/L. Moreover, the patient exhibited extreme hyperthyroidism with an iPTH of almost 3200 pg/mL 5 days prior to admission, declining to 2487 pg/mL after admission to our hospital. After treatment with high frequency and high flux dialysis (4 h hemodiafiltration with APS, four times per week), hyperbaric oxygen therapy (1.5 h per day), declining parathyroid hormone (1 μg of calcitriol injection every 2 days), and treatment for symptom relief (2 mg of haloperidol, 2 mg of clonazepam and 2 mg of benzhexol, administered orally twice per day), the clinical symptoms of the patient resolved gradually, with a decrease in both the frequency and amplitude of involuntary movement. Once the symptoms had achieved partial remission, follow-up MRI images acquired 14 days later showed high signal in the bilateral basal ganglia region on T2WI and T2FLAIR, which were nevertheless significantly weaker than the former MRI signal. About 1 month later, blood test results showed relatively stable uremic toxins (urea nitrogen 10.48 mmol/L, serum creatinine 641.5 μmol/L, uric acid 435 μmol/L, phosphorus 1.43 mmol/L, calcium 2.30 mmol/L,anion gap 15.9 mmol/L), and relieved hyperthyroidism (iPTH 1609 pg/mL). Finally, the patient achieved complete remission with normal body movement, and brain MRIs taken upon discharge showed an almost complete resolution of the lesions with slightly hyperintense signal on T2WI and T2FLAIR.

UE is an unfamiliar toxic metabolic encephalopathy with typical CT/MR neuroimaging showing bilateral vasogenic or cytotoxic edema in the cerebral cortex or basal ganglia region, producing a series of neurological abnormalities, ranging from mild attention loss to coma. UE with basal ganglia injury is uncommon, and generally occurs in Asian patients with DM. In our case, the patient had non-diabetic UE with typical bilateral basal ganglia lesions, and presented with involuntary movement. Recognizing this syndrome is of increasing importance, especially considering its diagnostic and prognostic implications.
